# Impact of sea ice transport on Beaufort Gyre liquid freshwater content

**DOI:** 10.1007/s00382-022-06615-4

**Published:** 2023-01-26

**Authors:** Sam B. Cornish, Morven Muilwijk, Jeffery R. Scott, Juliana M. Marson, Paul G. Myers, Wenhao Zhang, Qiang Wang, Yavor Kostov, Helen L. Johnson, John Marshall

**Affiliations:** 1 Department of Earth Sciences, University of Oxford, Oxford, UK; 2 Geophysical Institute, University of Bergen and Bjerknes Centre for Climate Research, Bergen, Norway; 3 Norwegian Polar Institute, Tromsø, Norway; 4 Massachusetts Institute of Technology, Boston, USA; 5 Department of Earth and Atmospheric Sciences, University of Alberta, Edmonton, Canada; 6 Centre for Earth Observation Science, University of Manitoba, Winnipeg, Canada; 7 Institute of Oceanology, Chinese Academy of Sciences, Qingdao, China; 8 Alfred Wegener Institute, Helmholtz Center for Polar and Marine Research (AWI), Bremerhaven, Germany; 9 College of Life and Environmental Science, University of Exeter, Exeter, UK

**Keywords:** Beaufort Gyre, Freshwater, Sea ice

## Abstract

The Arctic Ocean’s Beaufort Gyre (BG) is a wind-driven reservoir of relatively fresh seawater, situated beneath time-mean anticyclonic atmospheric circulation, and is covered by mobile pack ice for most of the year. Liquid freshwater accumulation in and expulsion from this gyre is of critical interest due to its potential to affect the Atlantic meridional overturning circulation and due to the importance of freshwater in modulating vertical fluxes of heat, nutrients and carbon in the ocean, and exchanges of heat and moisture with the atmosphere. Here, we investigate the hypothesis that wind-driven sea ice transport into/from the BG region influences the freshwater content of the gyre and its variability. To test this hypothesis, we use the results of a coordinated climate response function experiment with four ice-ocean models, in combination with targeted experiments using a regional setup of the MITgcm, in which we rotate the surface wind forcing vectors (thereby changing the ageostrophic component of these winds). Our results show that, via an effect on the net thermodynamic growth rate, anomalies in sea ice transport into the BG affect liquid freshwater adjustment. Specifically, increased ice import increases freshwater retention in the gyre, whereas ice export decreases freshwater in the gyre. Our results demonstrate that uncertainty in the ageostrophic component of surface winds, and in the dynamic sea ice response to these winds, has important implications for ice thermodynamics and freshwater. This sensitivity may explain some of the observed inter-model spread in simulations of Beaufort Gyre freshwater and its adjustment in response to wind forcing.

## Introduction

### The Beaufort Gyre and its freshwater content

The Beaufort Gyre is the largest reservoir of liquid freshwater content (FWC) in the Arctic Ocean (e.g. Haine et al. 2015; Carmack et al. 2016), storing a mean of 21,800 ${\hbox{km}}^3$ of FWC during 2003–2018 (Proshutinsky et al. 2019). Its ability to store, and potentially discharge, significant volumes of freshwater makes its dynamics of key interest for studies of the climate system (Lique et al. 2016). This is because large fluxes of freshwater from the Arctic to the North Atlantic have the potential to impact the global overturning circulation (e.g. Jahn and Holland 2013; Yang et al. 2016; Wang et al. 2018a; Holliday et al. 2020; Zhang et al. 2021), have been implicated in previous salinity anomalies in the subpolar North Atlantic (Dickson et al. 1988; Belkin et al. 1998), and are likely to increase in the future with climate change (Jahn and Laiho 2020).

The upper-ocean circulation of the Beaufort Gyre is sustained by the anticyclonic winds of the climatological Beaufort Sea High (Serreze and Barrett 2011; Timmermans and Marshall 2020), driving Ekman convergence and halocline deepening within the gyre (Proshutinsky et al. 2002). Hydrographic observations and satellite-derived measurements of sea surface height have revealed a substantial freshening of the Beaufort Gyre since the mid-1990s (Rabe et al. 2011; Giles et al. 2012; Krishfield et al. 2014; Proshutinsky et al. 2009), including an increase in FWC of 6400 ${\hbox{km}}^3$ during 2003–2018 (Proshutinsky et al. 2019); an increase of 40% relative to previous climatology. These changes are thought to have been driven by a combination of a relatively anticyclonic atmospheric circulation since the mid-1990s, increases in the surface anticyclonic stress due to a changing sea ice cover, the direct contribution of sea ice melt, and redirection of low salinity flows from the Mackenzie River and the Bering Strait by winds (Armitage et al. 2017; Giles et al. 2012; Proshutinsky et al. 2019; Wang et al. 2018b; Johnson et al. 2018). In tandem, the kinetic energy of the gyre has approximately doubled during the same period (Regan et al. 2019b; Armitage et al. 2017).

While winds over the Canada Basin vary on a range of timescales, the time-mean wind stress is anticyclonic and the resulting time-mean Ekman transport is convergent (e.g. Ma et al. 2017). One or more stabilising processes must thus exist to oppose perpetual Ekman-driven halocline deepening and freshwater accumulation (Manucharyan et al. 2016). Mesoscale eddies, activated by baroclinic instability, can act to flatten the slope of the halocline around a convergent lens (Marshall et al. 2002), and have been shown in eddy-resolving models to sustain a realistic Beaufort Gyre-like halocline (Manucharyan and Spall 2016). The halocline depth and equilibration timescales are then inversely related to the mesoscale eddy diffusivity (Davis et al. 2014; Manucharyan and Spall 2016). The other principal process opposing freshwater accumulation under anticyclonic winds arises from reductions (or reversals) of the anticyclonic ice-ocean shear. As the geostrophic circulation spins up, the relative stress imparted decreases (Zhong et al. 2018; Meneghello et al. 2018b; Wang et al. 2019). During winter, internal stresses within the pack ice slow ice motion, such that the ice may act as a drag on the surface ocean currents and impart cyclonic stress (Dewey et al. 2018; Meneghello et al. 2018b): a mechanism dubbed the *ice-ocean governor* (Meneghello et al. 2018a).

The steep continental slopes bordering the gyre stabilise halocline slopes, favouring stronger circulation, a deeper halocline, and prolonged equilibration timescale (Manucharyan and Isachsen 2019). However, to the north, the gyre is unconstrained by bathymetry, and is able to expand outwards—a mechanism which may help limit the build-up of eddy kinetic energy (Regan et al. 2019b). Observations support this idea that the gyre is able to expand geographically and that its centre may wander in response to atmospheric forcing (Regan et al. 2019a; Wang 2021).

As detailed above, much has been learnt in recent years about the dynamics of the Beaufort Gyre. As yet unexplored, however, is the role that sea ice may play as a means of redistributing freshwater into and out of the Beaufort Gyre.

### Hypothesis under consideration: ice transport impacts freshwater

In this paper, we seek to test the hypothesis that Beaufort Gyre liquid FWC is sensitive to the lateral volume fluxes (i.e., import/export) of sea ice into/out of the region. In particular, we hypothesise that net ice import into the gyre region should bolster Beaufort Gyre liquid FWC, while net ice export should act to drain liquid FWC. We investigate this hypothesis both under natural variability and under forced change (by sustained wind anomalies).

Sea ice growth rate is determined by a combination of thermodynamic and dynamic processes (Thorndike et al. 1975). Thermodynamic processes comprise freezing and melting, while dynamic thickness changes result from convergence via ridging and rafting, and thinning due to divergence through lead formation, which creates open water. Thermodynamic changes in sea ice thickness result in an exchange of freshwater with the ocean, owing to the comparatively low salinity of sea ice. By acting as a pathway between locations of freeze and melt, sea ice motion redistributes freshwater around the Arctic (Steele and Flato 2000). Sea ice motion can also change the local sea ice thickness through advection of thicker/thinner sea ice, and deformation of the ice pack. While these dynamic changes in themselves cause no exchange of freshwater with the ocean, they may alter the net thermodynamic growth rate—which is partly controlled by sea ice thickness—and thereby yield exchanges of freshwater with the ocean. We hypothesise that, via this connection between dynamic and thermodynamic sea ice thickness changes, wind-blown sea ice transport may impact liquid freshwater.

During winter, a negative feedback exists between sea ice thickness and thermodynamic growth rate of sea ice: thin ice grows faster than thick ice (Bitz and Roe 2004; Thorndike et al. 1975). In the absence of sunlight, the thermodynamic growth rate is determined by the balance of upwards heat fluxes to the base of the ice, and the conductive heat flux across the ice to the atmosphere. In the Canada Basin, oceanic heat fluxes to the underside of the ice are low, generally $<\,1\,\hbox {Wm}^{-2}$ (Shaw et al. 2009). As a result, the winter thermodynamic growth rate of sea ice is dominantly explained by the conductive heat loss through the ice and overlying snow; a flux that is inversely proportional to the thickness of both layers (e.g. Petrich and Eicken 2010). As such, we hypothesise that the winter export of ice from a region acts to drain the liquid reservoir of freshwater through dynamically thinning the ice pack, increasing the thermodynamic growth rate, transferring water from the liquid to the solid phase, and transporting that freshwater out of the region through sea ice export. During the summer melt season, these relationships may change, as thin sea ice can lead to more rapid melting by allowing earlier penetration of solar radiation into the mixed layer (Maykut and McPhee 1995; Perovich et al. 2008), thereby returning freshwater to the ocean at a faster rate. However, the amount of freshwater that can be returned to the ocean is limited by the thickness of the ice.

Mean dynamic ice thickness changes in a given region are related to the volume transport of ice across the region’s lateral boundaries by the divergence theorem. This ice import/export relies on the sea ice velocity field—specifically, either the strain in that velocity field or the way it advects ice of spatially non-uniform thickness. In this paper, we focus our attention on the transport of ice under a stationary sea-level pressure (SLP) pattern. In this scenario, whether ice converges into the regions or diverges, depends on the angle between ice flow and isobars. Note that there are other potential scenarios in which ice transport into/out from the region could occur.

**Fig. 1:**
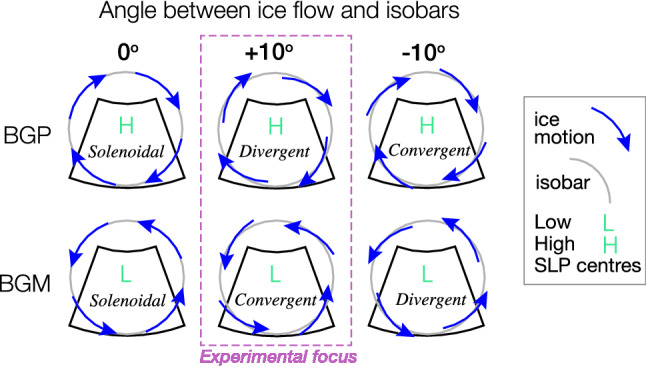
Sea ice may converge or diverge under a stationary SLP anomaly according to the cross-isobaric angle of flow. Diagram schematically depicts BG region, as indicated by black box. Blue arrows indicate ice motion. Whether sea ice flow is solenoidal, divergent, convergent is indicated in italics. Dashed magenta line outlines the scenarios we explore experimentally in Sect. 3.2. BGP stands for Beaufort Gyre Plus, the anticylonic wind anomaly experiment; BGM stands for Beaufort Gyre Minus, the cyclonic wind anomaly experiment; these experiments are introduced in Sect. 2.2

### Ageostrophic motion of atmospheric and oceanic boundary layers in presence of sea ice

In the atmospheric and oceanic boundary layers, friction breaks the geostrophic balance, causing flow to deviate from isobars. The observed flow, ${\mathbf {u}}$ is then comprised of a geostrophic component, $\mathbf {u_g}$, and an ageostrophic component, $\mathbf {u_a}$, as per ${\mathbf {u}} = \mathbf {u_g} + \mathbf {u_a}$. The ageostrophic or cross-isobaric flow in the frictional boundary layers determines the convergence and divergence of mass within these boundary layers: whether air, sea ice, or seawater. These turning angles are thus highly relevant for the freshwater dynamics of the Beaufort Gyre—given that freshwater is concentrated in the surface ocean and in sea ice. Variation in the angle between sea ice motion and isobars, and corresponding influence on divergence/convergence of sea ice is illustrated in Fig. 1. We now briefly review the controls on the angular relations between geostrophic winds, surface winds, and sea ice motion.

#### Cross-isobaric angle of surface wind

Surface wind is rotated anticlockwise relative to geostrophic wind in the northern hemisphere, and clockwise in the southern hemisphere. Observed turning angles show a considerable range: values of 6$^\circ$–45$^\circ$ are reported over land (e.g. Blackadar 1962; Lettau 1967; Mendenhall 1967; Hoxit 1973; Hess and Garratt 2002; Lindvall and Svensson 2019), and smaller values of 6$^\circ$–14$^\circ$ over the ocean (Gray 1972; Mendenhall 1967). There are fewer observations in the polar marine environment, but the drag coefficient of sea ice surfaces lies between those of the open ocean and most land surfaces (Andreas 1996; Overland 1985), implying intermediate values for the cross-isobaric angle over sea ice. This is dependent on the roughness and hence the age of the sea ice. The cross-isobaric angle of surface wind is known to change with several variables. In a neutral atmosphere, Rossby number (Ro) theory states that when the geostrophic wind speed, *G*, Coriolis parameter, *f*, and the roughness length, $z_0$, are known then the cross-isobaric angle and geostrophic drag coefficient can be calculated (e.g. Hess and Garratt 2002). With the surface Rossby number formulated as $\mathrm {Ro} \equiv G/(\vert {}f\vert {}z_0)$, the cross-isobaric angle should decrease as Ro increases (Blackadar 1962; Kung 1968). However, in non-neutral atmospheric cases, the isobaric angle shows a strong dependence on atmospheric stability (Lindvall and Svensson 2019; Van Ulden and Holtslag 1985). In the polar marine environment, there are important seasonal variations in *G*, $z_0$ (which is related to the drag coefficient; Overland 1985) and atmospheric stability, and potentially long-term trends associated with climate change. As a result, we might expect seasonal and long-term trends in the cross-isobaric angle of surface wind.

#### Air-ice turning angle

Sea ice velocities are primarily explained by the tractions applied on the top and bottom surfaces by the atmosphere and ocean, but also depend on Earth’s rotation, internal stresses within the ice, and the tilt of the sea surface (e.g. Thorndike and Colony 1982; McPhee 2012; Colony and Thorndike 1984). The turning angle of ice relative to surface winds (the *air-ice angle*) also varies substantially with these parameters (Park and Stewart 2016). While it is an oft-cited rule of thumb that sea ice in the northern hemisphere drifts at about 2% of the surface wind speed, and at an angle 30$^\circ$–45$^\circ$ to the right of the surface winds (Nansen 1902; Colony and Thorndike 1984; Rigor et al. 2002), observations show a wide range of air-ice angles, and a seasonal cycle with deviations further to the right of isobars in the summer (Thorndike and Colony 1982; Heorton et al. 2019; Cole et al. 2017).

#### Cross-isobaric angle of ice flow

The angle between ice motion and the geostrophic wind is determined by the sum of the cross-isobaric angle of surface wind and the air-ice turning angle. As (in the northern hemisphere) the former turns to the left, while the latter turns to the right, the cross-isobaric angle of ice flow tends to be relatively small, though it shows considerable spread in observations, as well as systematic spatial, seasonal, and long-term variability (Kimura and Wakatsuchi 2000; Maeda et al. 2020).

#### Ice-ocean boundary layer

The ice-ocean boundary layer exhibits similar frictional physics to the atmospheric boundary layer, though at a planetary scale that is roughly 30 times smaller (McPhee 2017). The ice concentration and roughness of the underside of the ice, the drift speed (itself related to ice properties), the stability of the water column, and the geostrophic velocities below again combine to help determine the complex flow pattern of the ice-ocean boundary layer and present major challenges for simulation efforts (McPhee and Smith 1976; McPhee 1981, 2012; Cole et al. 2014, 2017). Integrating over the frictional boundary layer in the upper ocean yields the Ekman transport; directed c. 90$^\circ$ to the right of the surface wind.

Climate models may directly solve for these turning angles across the boundary layers, rather than prescribing them (as per Hibler 1979). However, different bulk formulae exist and different vertical resolutions may affect how justified these solving schemes are (e.g. Hunke et al. 2011). Comparisons show that model solutions show important differences in turning angle statistics and often disagree markedly with observations, whether for sea ice drift (Girard et al. 2009; Martin and Gerdes 2007) or the cross-isobaric angle of surface wind (Lindvall and Svensson 2019; Svensson and Holtslag 2009).

## Methods

### CRF method: background

In this study, we use a model perturbation method known as a Climate Response Function (CRF) experiment (Marshall et al. 2017). A CRF experiment involves observing how a model responds to an abrupt and sustained change in some aspect of model forcing, through a comparison to an unperturbed control run. CRF experiments have proven useful in cleanly delineating relationships in simulated climate systems, including responses to changes in: the concentration of greenhouse gases, ozone or aerosols; wind patterns; river or glacial meltwater runoff (e.g. Good et al. 2011, 2013; Marshall et al. 2014; Lambert et al. 2019; Muilwijk et al. 2019). The CRF (or *step* response function) is the time integral of the characteristic *impulse* response function. The latter can be convolved with a time history of the forcing to estimate a time history of the response, as per linear response theory (e.g. Hasselmann et al. 1993). Linear response functions can also be derived directly from long control runs of fully coupled models (e.g. Kostov et al. 2017), and convolved with observationally-derived forcing timeseries in order to compare against nature (Johnson et al. 2018; Cornish et al. 2020). In model intercomparisons, climate response functions offer a means to go beyond comparing mean states and benchmark across models how climate variables respond to specific forcings. These comparisons provide the opportunity to identify the underlying causes of these differences, assisting both model development and conceptual understanding.

In this study, we rely on coordinated CRF experiments designed by Marshall et al. (2017) to probe the behaviour of key circulation systems in the Arctic Ocean; an effort in collaboration with modelling groups under the auspices of the Forum for Arctic Modelling and Observational Synthesis (FAMOS; Proshutinsky et al. 2016). The first such coordinated series of experiments is presented and analysed by Muilwijk et al. (2019), and focuses on ocean and sea ice responses to changes in the strength of the Greenland Sea Low. Here, we present results from experiments designed to probe the response of the Beaufort Gyre to abrupt changes in the wind associated with the strength of the Beaufort Sea High (Marshall et al. 2017). We require additional climate variables to those proposed by Marshall et al. (2017) and rely on the output from CRF experiments conducted by four contributing modeling groups.

### Experimental setup

**Fig. 2:**
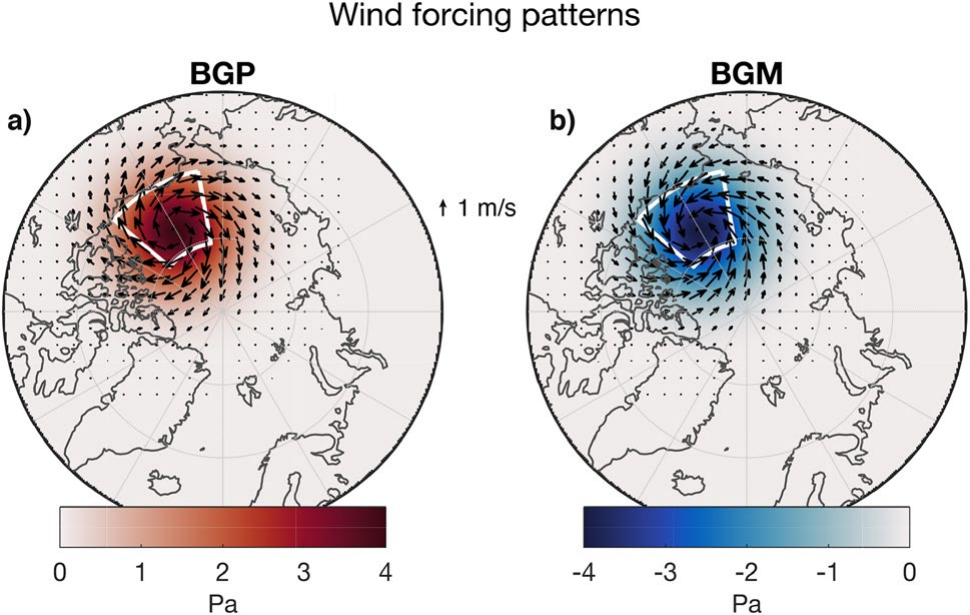
Wind forcing patterns used in the CRF experiments and their associated SLP patterns. **a** Beaufort Gyre Plus (BGP), an anticyclonic pattern that strengthens the Beaufort Sea High when applied to forcing fields; **b** Beaufort Gyre Minus (BGM), a cyclonic pattern that weakens the Beaufort Sea High when applied to forcing fields. White box outlines the BG study region

The experimental procedure involved perturbing the selected ice-ocean models with an identical 10 m wind anomaly, which was added to the original forcing fields (which vary from model to model). Two anomaly patterns were used: an anticyclonic pattern based on a strengthened Beaufort Sea High (BGP, for Beaufort Gyre Plus; Fig. 2a), and a cyclonic pattern based on a weakened Beaufort Sea High (BGM, for Beaufort Gyre Minus; Fig. 2b). The anomalies are centred on 77 $^\circ$N, 149 $^\circ$W, with a radius of influence on the order of 1000 km. This choice of location was based on an analysis of the modes and magnitudes of variability (Marshall et al. 2017) in sea-level pressure in the 1948–2015 6-hourly NCAR-NCEP atmospheric reanalysis (Kalnay et al. 1996). The peak magnitude of the perturbations is 4 hPa, a magnitude chosen to be representative of multi-year to decadal trends. The 10 m winds were then computed using the relation to the geostrophic wind in Eq. 1, following Proshutinsky and Johnson (1997):

1 $$\begin{aligned} W_{{\mathrm {s}}} = 0.7 \times \begin{bmatrix} \mathrm {cos}30 &{}\quad -\,\mathrm {sin}30 \\ \mathrm {sin}30 &{}\quad \mathrm {cos}30 \\ \end{bmatrix} \times W_{{\mathrm {g}}} \end{aligned}$$

As described in Sect. 1.3, the cross-isobaric angle and speed of the 10 m wind can be expected to vary in space and time in nature according to a range of factors. The choice of a specific, arbitrary cross-isobaric angle (30$^\circ$) eliminates this uncertainty in the CRF experiments. Whether ice converges or diverges in the anomaly under this pattern depends on the air-ice turning angle (consider Sect. 1.3 and Fig. 1. The surface wind anomalies and associated SLP patterns are shown in Fig. 2.

Control runs were archived after some initial “spin-up” time (Marshall et al. 2017). The perturbations were then applied abruptly to the control runs and sustained, in parallel, for 30 years. The CRFs are then determined by the anomalies in the perturbed run relative to the control run.

In all models, we compute diagnostics within a bounding box spanning 130 $^\circ$W–170 $^\circ$W and 70.5 $^\circ$N–80.5 $^\circ$N (see outline in Fig. 2). This area definition corresponds closely to that used in observational studies (Proshutinsky et al. 2009, 2019). There are drawbacks to this definition, however—not all forced ice-ocean models show a gyre that is tightly constrained within this area (Wang et al. 2016). Indeed observations and models suggest that the gyre can expand toward the interior of the Arctic Ocean (Regan et al. 2019a, b). Nonetheless, the centre of the perturbation pattern lies within this region and therefore the bounding box captures the centre of action.

The principal diagnostics that we use and present here are: (1) total freshwater content, as

2 $$\begin{aligned} \mathrm {FWC}(t) = \iiint _{z(S=S_{\mathrm {ref}})}^{0} \frac{S_{\mathrm {ref}} - S}{S_{\mathrm {ref}}} {\mathrm {d}}V \end{aligned}$$

measured relative to a reference salinity ($\hbox {S}_{\mathrm {ref}}$) of 34.8, as per Aagaard and Carmack (1989; 2) sea ice volume; (3) sea ice volume transport to/from the region. For the latter two diagnostics, snow is included after conversion to a sea ice volume equivalent using a conversion factor of 330 kg $\hbox {m}^{-3}$/910 kg $\hbox {m}^{-3}$, according to the assumed densities of snow and ice, respectively.

### Participating models

Basic information about the participating models is shown in Table 1. The University of Alberta group contributed results from a regional version of NEMO (Madec and NEMO System Team 2008); the Alfred Wegner Institute (AWI) group contributed simulations with the unstructured mesh model FESOM (Wang et al. 2014; Danilov et al. 2015); the MIT group contributed a regional configuration of MITgcm (Marshall et al. 1997); and the University of Bergen (UiB) group contributed simulations from NorESM (Bentsen et al. 2012).

The models are all ice-ocean models, with prescribed atmospheric forcing, including near-surface winds, air temperatures, humidity, downward longwave and shortwave radiation, and precipitation (see Table 1). The Alberta NEMO model uses a repeated 2002–2016 forcing and FESOM uses a “normal year” forcing. Because 30 years are required for the CRF comparisons, these shorter atmospheric forcing datasets must be repeated. The MITgcm and NorESM runs are forced by atmospheric reanalyses that exceed 30 years in length, and the wind anomalies were applied in January 1980 in both cases.

In their control simulations, the models receive surface winds from their respective forcing datasets. As such, the uncertainty in the cross-isobaric angle of surface winds is “outsourced” to the reanalysis product or climatology that they use to provide these fields. However, the turning of the ice beneath these surface winds, and the dynamics of the ice-ocean boundary layer, are simulated by the models. All models use a 0$^\circ$ turning angle in the ice-ocean stress. The implicit assumption in the use of this zero value is that the upper ocean vertical resolution is sufficient to resolve the dynamics of the ice-ocean boundary layer, including the Ekman spiral, without needing a prescribed adjustment (Hunke et al. 2011). The thickness of the uppermost ocean grid cell is listed in Table 1 and gives an indication of whether this assumption is fair.

**Table 1:** Information about participating models

Group	Ocean model	Ice model	Horizontal res.	Domain/grid	Forcing	Top ocean cell thickness (m)	*S* restoring
Alberta	NEMOv3.4	LIM 2	Nominal 0.5$^\circ$	Regional/tripolar ORCA05	CGRF (Smith et al. 2014)	1.0225	No
AWI	FESOM	FESIM	25 km Arctic	Global/unstructured	CORE normal yr. (Griffies et al. 2012)	10	No
MIT	MITgcm	MITgcm	36 km	Regional/cubedsphere	JRA-25 (Onogi et al. 2007)	5	No
UiB	NorESM-O	CICE 4	Nominal 1$^\circ$	Global/tripolar	20CR (He et al. 2016)	2.5–10	Yes

### Modified wind experiments with MITgcm

In order to directly test the sensitivity of freshwater accumulation in the Beaufort Gyre to wind-driven ice export/import, we perform dedicated experiments with the MITgcm. We seek to actively perturb sea ice trajectories—and therefore sea ice import into the gyre region—by modifying the surface wind forcing, and investigating the cascade of downward impacts.

We perform two parallel sets of experiments. Firstly, the original CRF experiment, as per Sect. 2.2, which is comprised of the model runs CTRL, BGP, and BGM. Secondly, a CRF experiment with a rotation of wind vectors by 10$^\circ$ anticlockwise only over sea ice. The modification is applied to both the background winds and the perturbation winds, and yields the runs CTRL10, BGP10, and BGM10. The 10$^\circ$ angle is considered to roughly reflect the uncertainty in the cross-isobaric angle of surface wind over sea ice. Only one direction of rotation to the wind vectors is required in order to probe both ice export and import scenarios, because it is applied to both anticyclonic and cyclonic wind anomalies (see Fig. 1). We isolate the effect of ice import or export by comparing against the original perturbation experiments. CRFs are calculated using the respective control and perturbation runs for each experiment, described in Sect. 2.2.

**Table 2:** Reference table for experiment names. BGP CRF and BGM CRF are computed for all participating models. The rest are computed in the experiments with MITgcm only. BGP is ‘Beaufort Gyre Plus’, the wind-forcing pattern corresponding to a strong Beaufort Sea High (Fig. 2a). BGM is ‘Beaufort Gyre Minus’, the wind-forcing pattern corresponding to a weakened Beaufort Sea High (Fig. 2b). CTRL is ‘control’

Name in text	Calculated as	Appears in figure
BGP CRF	BGP − CTRL	3a, c and 6a
BGM CRF	BGM − CTRL	3b, d and 6c
CTRL10-CTRL	CTRL10 − CTRL	5
BGP10 CRF	BGP10 − CTRL10	6b
BGM10 CRF	BGM10 − CTRL10	6d
EXP	BGP10 − CTRL10 − (BGP − CTRL)	7
IMP	BGM10 − CTRL10 − (BGM − CTRL)	8

Note that in calculating the CRFs we explicitly separate these two model configurations: the control simulation for calculating the BGP10 and BGM10 CRFs is a control simulation with a 10$^\circ$ rotation of surface winds over sea ice (Table 2). As such, the BGP10 CRF represents the modeled response to the BGP wind forcing pattern in a model Arctic with surface winds (including the anomalous winds) rotated 10$^\circ$ anticlockwise of the original model solution for surface winds. See Table 2 for details of the experiment names.

We examine additional model diagnostics in the MITgcm (Table 3). As well as (1) freshwater content, (2) sea ice volume and (3) sea ice export, we also analyse: (4) liquid freshwater fluxes, (5) ice-to-ocean freshwater fluxes from melting and freezing, (6, 7) precipitation minus evaporation on ice and ocean, respectively, and (8) sea ice thickness. Diagnostics (2, 3, 5–7) permit the construction of a complete freshwater budget for the ice component, while (8) allows us to see how the ice export/import relates to thickness changes. While diagnostics 6 and 7—precipitation on ice and ocean—were significant in the control run freshwater balance, they exhibit very little change in anomaly runs, and so we do not present them here. We include liquid freshwater fluxes (4) in order to understand whether modifying the cross-isobaric angle of surface wind affects liquid freshwater accumulation directly via Ekman transport.

**Table 3:** Acronyms for climate variables as displayed in figures

Acronym	Meaning	Sign convention
FWC	Liquid freshwater content relative to $S_{\mathrm {ref}}=34.8$	
IVOL	Sea ice volume	
IVOLF	Cumulative lateral sea ice volume flux (import/export)	Import is positive, export is negative
FWF	Cumulative lateral liquid freshwater flux above $S_{\mathrm {ref}}$ isohaline	Inflow is positive, outflow is negative
IOF	Cumulative ice-to-ocean freshwater flux (melting/freezing)	Melting is positive, freezing is negative
SIT	Sea ice thickness	

## Results

### Coordinated CRF experiments

**Fig. 3:**
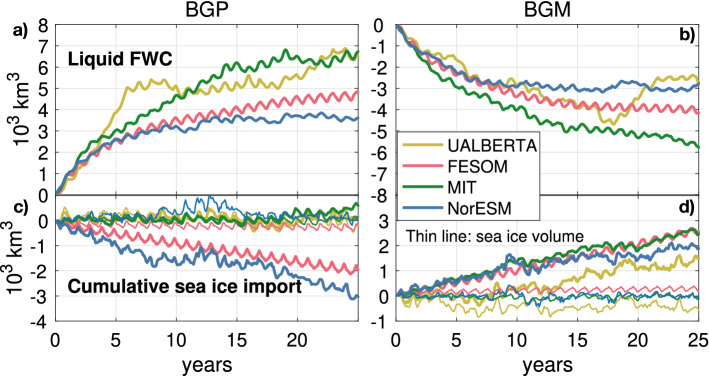
Climate response functions for BGP (left) and BGM (right) forcing patterns in four participating models. Liquid FWC responses shown in **a**, **b**. Cumulative sea ice import responses shown in **c**, **d**. Thin lines indicate sea ice volume responses. The SLP and associated surface wind forcing patterns are shown in Fig. 2

In Fig. 3 we present the CRFs for the four participating models (see Sect. 2.3). Under the anticyclonic BGP wind forcing, corresponding to an intensified Beaufort High, all models show an accumulation of liquid FWC (Fig. 3a), while under the cyclonic wind forcing (BGM), the models show a flushing of liquid FWC from the Beaufort Gyre (Fig. 3b).

In both the BGP and BGM CRFs, the initial adjustment in the first 4 years is very similar across the models. The overall timescale of adjustment is also similar between models. In both BGP and BGM, there is approximately a factor 2 difference between the maximum and minimum FWC anomalies at the end of 25 years. There are various possible explanations for the differences between the CRFs from each model. Likely important are different realisations of processes thought to dominate the freshwater balance in the gyre: Ekman pumping (despite a common surface wind anomaly), eddy-mediated isopycnal slumping, and effective ice-ocean governor strength. Here, however, we focus on the potential role of sea ice transport in explaining some of the observed differences (Fig. 3c, d).

Two models show approximately no response in ice volume import under BGP: the Alberta NEMO model and the MITgcm (Fig. 3c, green and yellow lines), while the other two show ice export from the region (blue and pink lines). The cumulative sea ice export reaches 2000 ${\hbox{km}}^3$ in FESOM and 3000 ${\hbox{km}}^3$ in NorESM by the end of the 25 year comparison period. Meanwhile, FESOM and NorESM show muted responses in sea ice volume within the region (as do all models; thin lines in Fig. 3c). By mass conservation, the difference between the sea ice volume anomaly and the anomaly in cumulative sea ice export must be balanced by sea ice growth. The two models showing sea ice export in the CRFs for BGP (NorESM and FESOM) also show lower liquid FWC accumulation than the other two models. This is consistent with our hypothesis, and suggests that the sea ice transport may—at least in part—explain the differences between simulated wind-driven Beaufort Gyre FWC accumulation.

All models show some ice import into the gyre region under the BGM wind forcing pattern. The Alberta model shows a muted response in the first ten years before exhibiting a similar rate of import to the other models, which show remarkably similar accumulations of ice within the region. Again, all models show a muted response in sea ice volume (the Alberta model shows a reduction, but this never exceeds 1000 ${\hbox{km}}^3$). The difference between accumulated sea ice import and sea ice volume changes in these models can be reconciled by an overall reduction in net thermodynamic growth rate. This is in line with the operation of a stabilizing feedback of sea ice thickness on growth rate. A reduction in sea ice growth rate implies a relative gain in liquid freshwater. In the case of BGM, this would result in a weaker freshwater loss. However, the differences in ice import across the models are small, and so we cannot expect this to be a significant source of variability in the FWC CRFs. Indeed, we see no obvious link between FWC and sea ice import in the BGM CRFs. Nonetheless, the relative stability of the ice volume timeseries (thin lines) is evidence that the hypothesised mechanism linking sea ice import and freshwater gain is at play. This relative gain in FWC may help to explain why the magnitude of freshwater loss in the BGM CRFs is generally smaller than the freshwater gain in the BGP CRFs—and not a perfect mirror image.

### Modified wind experiments

We now perform a stronger test of our hypothesis using a series of targeted experiments with one model, the MITgcm. We seek to isolate the effect of sea ice import/export, as described in Sect. 2.4.

We begin by describing the MITgcm control run (CTRL) climatology, and examine how an angular modification to the surface winds changes that climatology. Next, we investigate the system under forced change: we compare the results of the standard CRF experiments as described in Sect. 2.2, to those with an angular modification to the surface winds (Sect. 2.4). We explore the way that sea ice dynamics affect sea ice thermodynamics, and how this varies spatially and seasonally, in order to explain the overall effect on freshwater changes in the gyre.

#### The MITgcm CTRL and CTRL10 runs

**Fig. 4:**
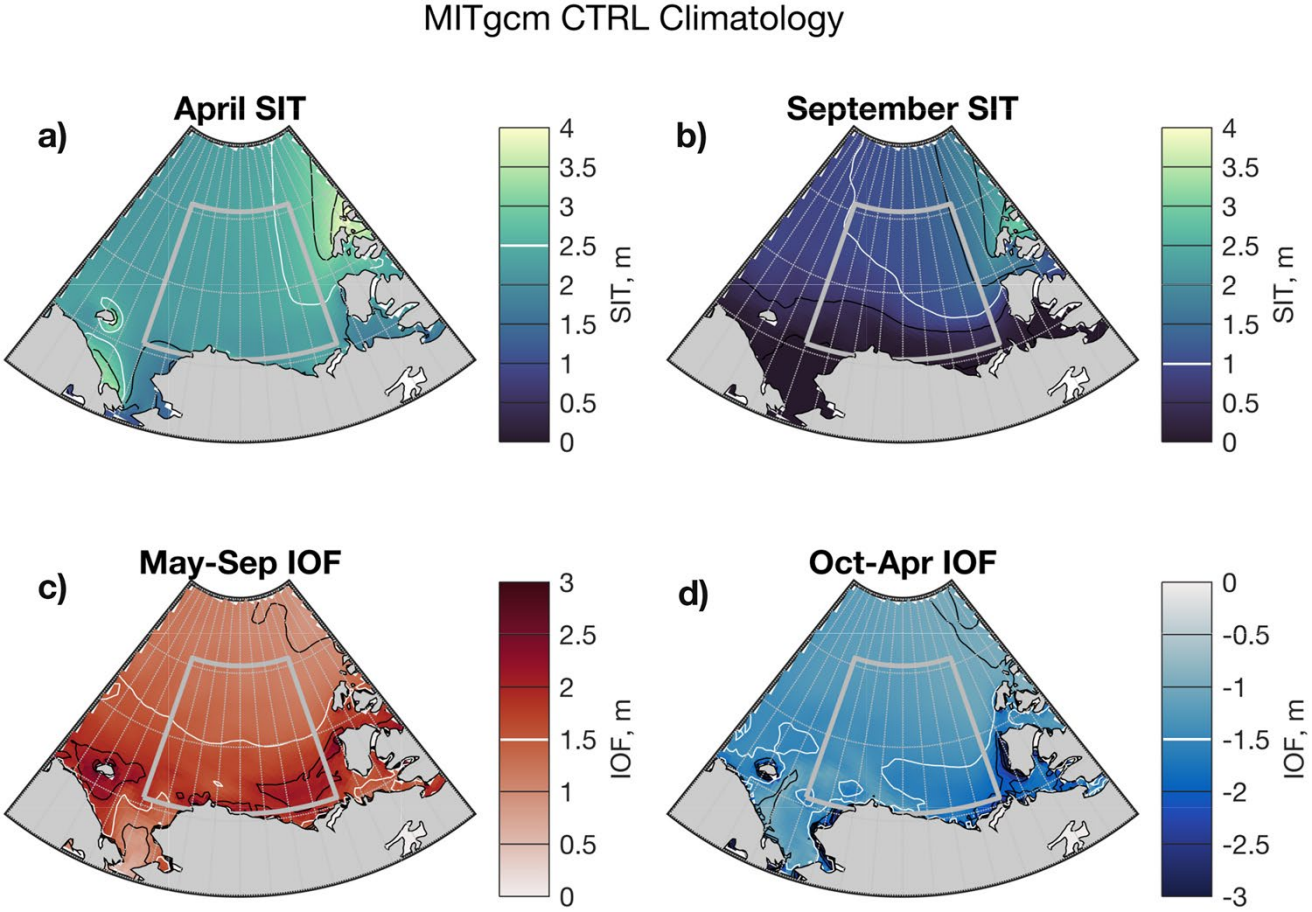
Mean control run (CTRL) fields from the MITgcm: sea ice thickness (SIT) in April (**a**) and September (**b**); thermodynamic ice-ocean freshwater fluxes (IOF) during summer (**c**) and winter (**d**). The contour that passes most centrally through the region in each case is highlighted in white to aid interpretation of scale

In the MITgcm control run (CTRL), sea ice at the end of winter has a mean thickness of 2–3 m in the gyre region (Fig. 4a), with ice thickening towards the Canadian Arctic Archipelago in the east. At the end of summer, the mean ice thickness approaches zero in the southern portion of the gyre region, but is 1–2 m thick in the north and east (Fig. 4b). This change in sea ice thickness over the summer involves a flux of freshwater to the ocean: Fig. 4c shows how these summertime freshwater fluxes are more intense (2–3 m) in the southern portion of the gyre region where the ice thickness decreases the most. In the winter, ice thickness is restored by a freshwater flux from the ocean into the ice (Fig. 4d). In addition to these thermodynamic changes in Fig. 4c, d, there are also dynamic contributions to sea ice thickness changes, and the spatial configuration of sea ice thickness.

**Fig. 5:**
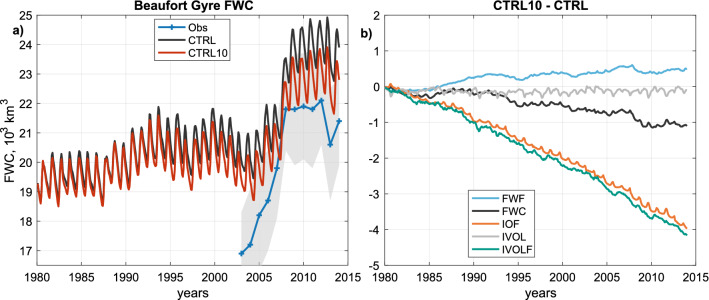
**a** Liquid freshwater content timeseries in the MITgcm CTRL run (black) and CTRL10 run (red). Observational estimates of FWC in the same region (blue, uncertainty shown by grey shading) from Proshutinsky et al. (2009, 2019). **b** Difference between diagnostics in the CTRL10 run and corresponding variables in the CTRL run. Flux terms are integrated through time. Acronyms are defined in Table 3

The integrated FWC within the gyre region is shown in Fig. 5a, in black for CTRL and red for CTRL10 (described in Sect. 2.4). We also show observational estimates of the FWC in the same region from Proshutinsky et al. (2009, 2019). A striking feature of both the CTRL run and the observational estimates is the marked increase in FWC that occurs 2005–2010 in the MITgcm and 2003–2010 in the observations (note that observations show further FWC increases after the end of the model run in 2014; Proshutinsky et al. 2019). As described in Sect. 1.1, this inflation of the gyre is thought to be mainly due to increasing anticyclonic wind stress, partly a result of atmospheric changes, and partly a result of changes to the pack ice that resulted in a more efficient transfer of momentum from winds to the ocean (e.g. Proshutinsky et al. 2009, 2019; Giles et al. 2012; Wang et al. 2018b). The MITgcm captures a considerable part of this FWC accumulation, but appears to slightly underestimate the increase in the earliest few years of the observational period presented; meanwhile, the absolute magnitude of FWC is higher in the MITgcm than in the observational estimates. Note that the simulation CTRL10 is not an attempt to improve the fit to the observational data—it is used solely for the purposes of investigating processes.

Comparing the MITgcm CTRL and CTRL10 runs highlights the impact of the 10$^\circ$ anticlockwise rotation of surface winds over sea ice in CTRL10 on sea ice and FWC in the BG. Over the CTRL10 run, a clear difference in FWC emerges relative to CTRL, which grows approximately linearly (black line, Fig. 5b), reaching ${-}\,1000 \,{\hbox{km}}^3$ by the end of the run. Some of the other key variables can help to explain this relative loss of FWC. There is an approximately constant flux of ice volume out of the gyre region in CTRL10 versus CTRL (teal line, Fig. 5b); indeed, the 10$^\circ$ anticlockwise rotation in CTRL10 should lead to a more divergent ice flow field under the anticyclonic winds that dominate the region. In absolute terms, the gyre is a net importer of sea ice in CTRL, and a net exporter in CTRL10; over the whole run, sea ice transport into the region reaches c. ${+}\,2000\,{\hbox{km}}^3$ in CTRL and c. ${-}\,2200\,{\hbox{km}}^3$ in CTRL10 (not shown). This difference in sea ice transport is closely matched by a commensurate increase in net thermodynamic growth rate (Fig. 5b, orange line), which leaves the sea ice volume reservoir in the gyre region relatively unaltered (Fig. 5b, grey line). The negative ice-to-ocean flux, in turn, drains the liquid freshwater reservoir. There is a small positive contribution to the FWC in CTRL10 versus CTRL from an increase in the liquid freshwater flux across the region’s boundaries (Fig. 5b, cyan line); however, the net effect acts to deplete the FWC reservoir (black line).

The 1000 ${\hbox{km}}^3$ FWC difference between CTRL and CTRL10 by the end of the run is a similar magnitude to the uncertainty in the observational estimates of FWC (Fig. 5a). As such, it is of a magnitude that might go undetected, but is still considerable, especially when considering differences across models (see Sect. 3.1).

#### Anomaly experiments

*Spatially integrated changes*

**Fig. 6:**
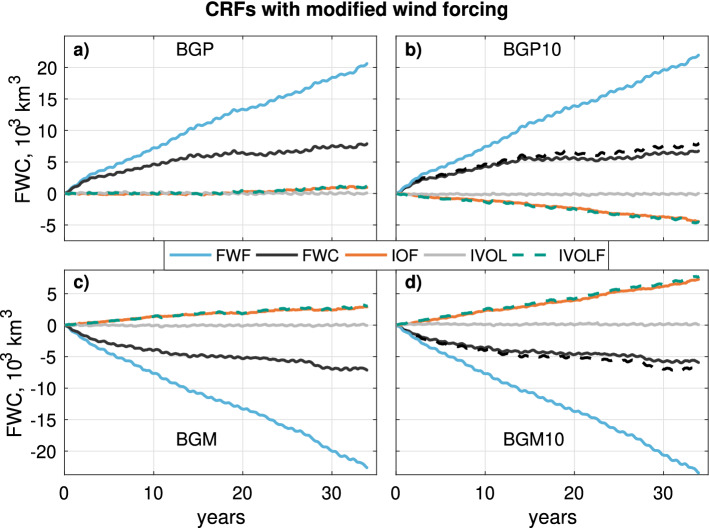
CRFs for different FWC budget terms in the regular CRF experiment (left side) and with + 10$^\circ$ wind forcing modification (right side). Fluxes are positive when directed into the gyre. Flux terms are integrated through time, so have units of FWC. BGM and BGP FWC curves are overlain on BGM10 and BGP10 plots as dashed black lines for ease of comparison. Acronyms for climate variables are defined in Table 3

We now investigate the response of Beaufort Gyre FWC to wind forcing, and the hypothesised role of sea ice transport in influencing this response. Fig. 6 shows the responses to imposed anticyclonic ‘BGP’ and cyclonic ‘BGM’ winds, in the normal model configuration, and in the configuration with a 10$^\circ$ anticlockwise rotation of the surface winds over sea ice. Importantly, the BGP10 and BGM10 CRFs are defined relative to the CTRL10 run (Table 2).

In the BGP CRF experiment, the imposed anticyclonic winds (Fig. 2a) induce a time-dependent accumulation of FWC up to a total of c. 8000 ${\hbox{km}}^3$ after 34 years (Fig. 6a, black line). The increase in FWC is clearly driven by the cumulative liquid freshwater flux (FWF) into this region (Fig. 6a, cyan line). The fact that the response curve approaches an equilibrium with time demonstrates that there must be one or more negative feedback processes (or ‘sinks’) that are dependent on the volume of freshwater in the gyre (Manucharyan et al. 2016; Doddridge et al. 2019); note how the source of FWC due to FWF clearly outstrips the FWC response. The subtle decrease in the slope of the cyan line during the first 5 years most likely reflects the damping effect of the ice-ocean governor: as the geostrophic circulation spins up, the relative anticylonic stress imparted by the ice decreases, leading to a decrease in the convergent Ekman transport of liquid freshwater (Meneghello et al. 2018a), detectable in the FWF term (Fig. 6). The timescale for this adjustment is known to be significantly shorter than that mediated by baroclinic eddies (Doddridge et al. 2019). There is almost no response of ice import in the BGP CRF; the ice velocity anomalies follow a solenoidal trajectory (not shown) as schematised in Fig. 1 (upper left hand panel).

The BGP10 CRF (see Sect. 2.4 for definition), on the other hand, exhibits roughly linear cumulative sea ice export, reaching ${-}\,5000 \,{\hbox{km}}^3$ after 34 years (Fig. 6b, dashed teal line). A more divergent sea ice flow field is expected under an anticlockwise modification of an anticyclonic flow pattern (consider Fig. 1, upper central panel). This ice export is compensated by a commensurate flux of freshwater from the ocean to ice (orange line), which buffers the ice volume in the region, keeping it at a steady (though non-zero) level (grey line). A small thickness anomaly is required to drive anomalies in thermodynamic growth rate, as we show later in this subsection. Relative to the BGP CRF, the FWC accumulation in the BGP10 CRF is reduced by 15%, or 1175 km $^3$ (after 34 years). Given that the liquid freshwater fluxes into the region in the BGP10 CRF are actually slightly higher than in the BGP CRF (compare the cyan lines in Fig. 6a, b), the most plausible explanation for the lower FWC accumulation is the draining of the liquid freshwater reservoir by sea ice export and attendant increase in sea ice growth.

In the BGM CRF experiment, a cyclonic wind pattern is applied (Fig. 2b), designed to promote the flushing of the gyre. The BGM freshwater content CRF is close to a mirror image of the BGP equivalent, and shows a time-dependent decrease of FWC that slows over time, reaching ${-}\,7200\,{\hbox{km}}^3$ after 34 years (Fig. 6c, black line). This flushing is driven by anomalous liquid freshwater export through the region’s boundaries (cyan line). This outwards freshwater flux is offset to a small extent by ice import (dashed teal line) and a commensurate slowing of the net thermodynamic growth rate (orange line), leading to an input of freshwater into the liquid phase. The flattening of the FWC CRF curve represents the gradual slowing of the FWC-dependent sink process relative to the CTRL run, in which FWC levels are relatively elevated.

The BGM10 CRF exhibits a greater rate of ice import into the region (Fig. 6c, dashed teal line) than in the BGM CRF. A more convergent ice flow field is expected with an anticlockwise modification of a cyclonic flow pattern (consider Fig. 1, lower central panel). The anomalous ice import is compensated by a net flux of freshwater from ice to ocean (Fig. 6c, orange line). The lateral liquid freshwater flux (cyan line) is relatively unchanged versus the BGM CRF. The net effect of these processes is to bolster the FWC in the BGM10 CRF relative to the BGM CRF (black line versus black dashed line) by 17%, or 1230 ${\hbox{km}}^3$ (after 34 years).

Relative to the BGP and BGM CRFs, the BGP10 and BGM10 CRFs exhibit significant anomalies in ice-ocean freshwater fluxes (dashed teal lines). These exceed the resulting anomalies in FWC. The lower magnitude of change in FWC is most likely due to the fact that the sink terms of FWC are FWC-dependent (Manucharyan et al. 2016); any change in constant flux terms into or from the gyre will be buffered by the adjustment of the FWC-dependent sink terms.

The results show that, by changing the ice import into the gyre region, liquid FWC can be affected via a feedback between ice volume and thermodynamic exchange of freshwater between the ice and ocean. We now examine how this relationship between dynamic and thermodynamic changes in sea ice manifests spatially and seasonally, in order to understand the spatially integrated signal in Fig. 6.

*Spatial and seasonal changes*

**Fig. 7:**
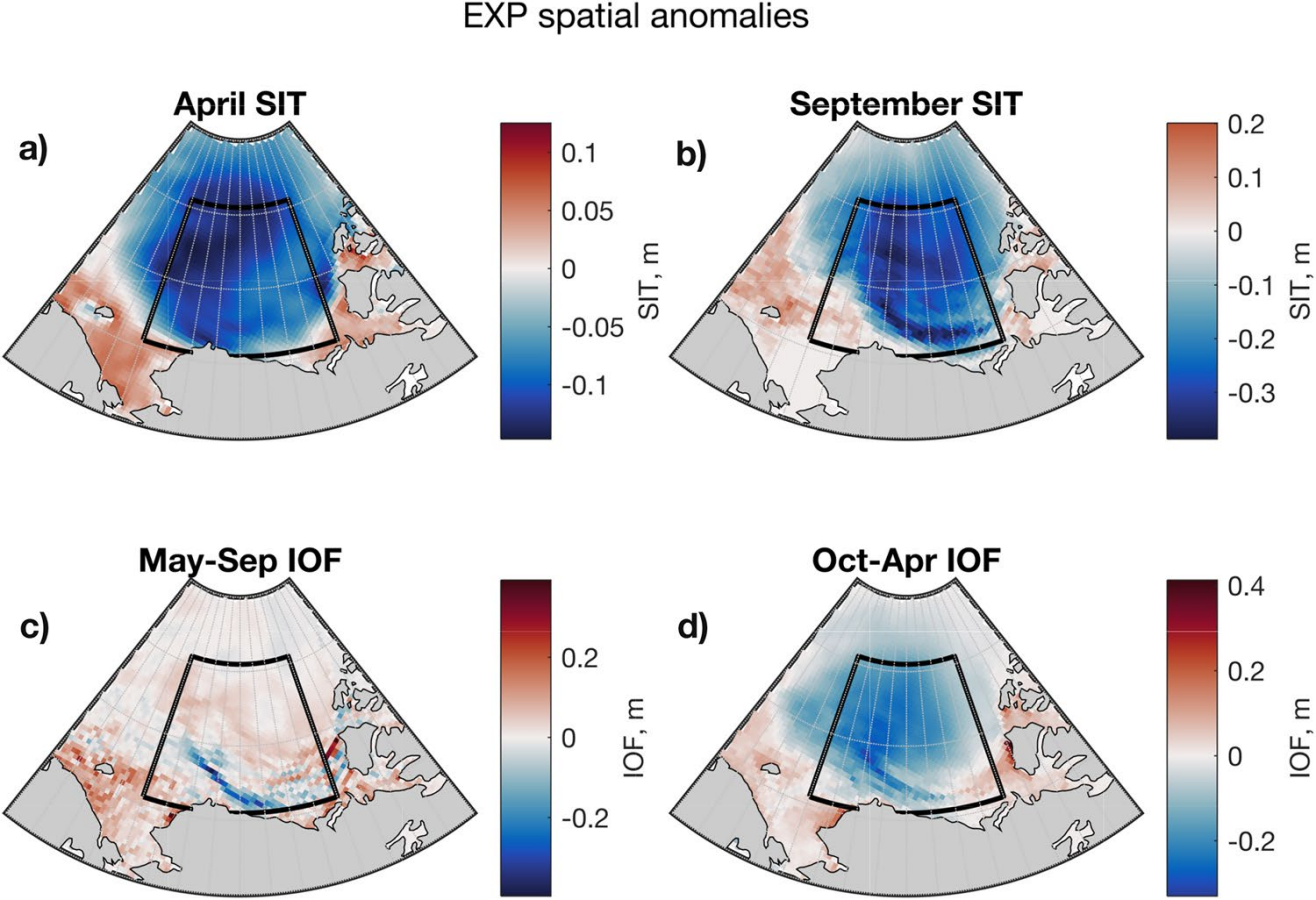
Mean seasonal evolution of anomalies in ice thickness and ice thermodynamics in EXP (defined in Table 2). Blue colours in **a**, **b** denote thinned sea ice cover in April and September, respectively. Red colours in **c** indicate increased summertime melting, while blue colours in **d** show increased wintertime freezing

As described above, the main differences between the BGP10 CRFs and BGP CRFs are driven by the additional sea ice export in the BGP10 CRF. We can isolate the effect of the elevated ice export by taking the difference between these sets of anomalies, yielding results we refer to as EXP (Table 2). In Fig. 7, we show the seasonal evolution of anomalies in sea ice thickness and ice-ocean fluxes under EXP. The sea ice export yields persistently low sea ice thickness anomalies in the gyre region in both summer and winter (Fig. 7). Note that this persistent thickness anomaly is related to persistent, but small, differences in sea ice volume (Fig. 6).

In the summer period, the low sea ice thickness promotes melting across the central, northern, and eastern parts of the region. Observations show that thin sea ice gives way to open water sooner than thick ice, allowing earlier solar heating of the mixed layer, which increases SSTs (Steele and Dickinson 2016) and drives basal melting of ice (Perovich et al. 2011, 2008). We expect these effects to be captured in our simulations, explaining the enhanced melting in the interior part of the gyre region (Fig. 7c). In the southern part of the region, the dynamic thinning preconditions a reduction in melting, because these are regions where little ice survives the summer (Fig. 4b), and thus reduced ice thickness equates to reduced freshwater availability.

In the winter period, on the other hand, the dynamic thinning yields an increased freshwater flux from the ocean to sea ice—an increase in the net thermodynamic growth rate of ice, due to the inverse dependence of growth rate on sea ice thickness (e.g. Petrich and Eicken 2010). There is a close spatial match ($R=0.70$ in the plotted region) between the mean September sea ice thickness anomaly pattern and the mean winter ice-ocean freshwater flux anomaly (compare Fig. 7b, d). Note that the strength of this correlation is bound to be limited due to advection of the uneven sea ice thickness field during the winter (after September). This strong winter signal dominates the overall freshwater response.

**Fig. 8:**
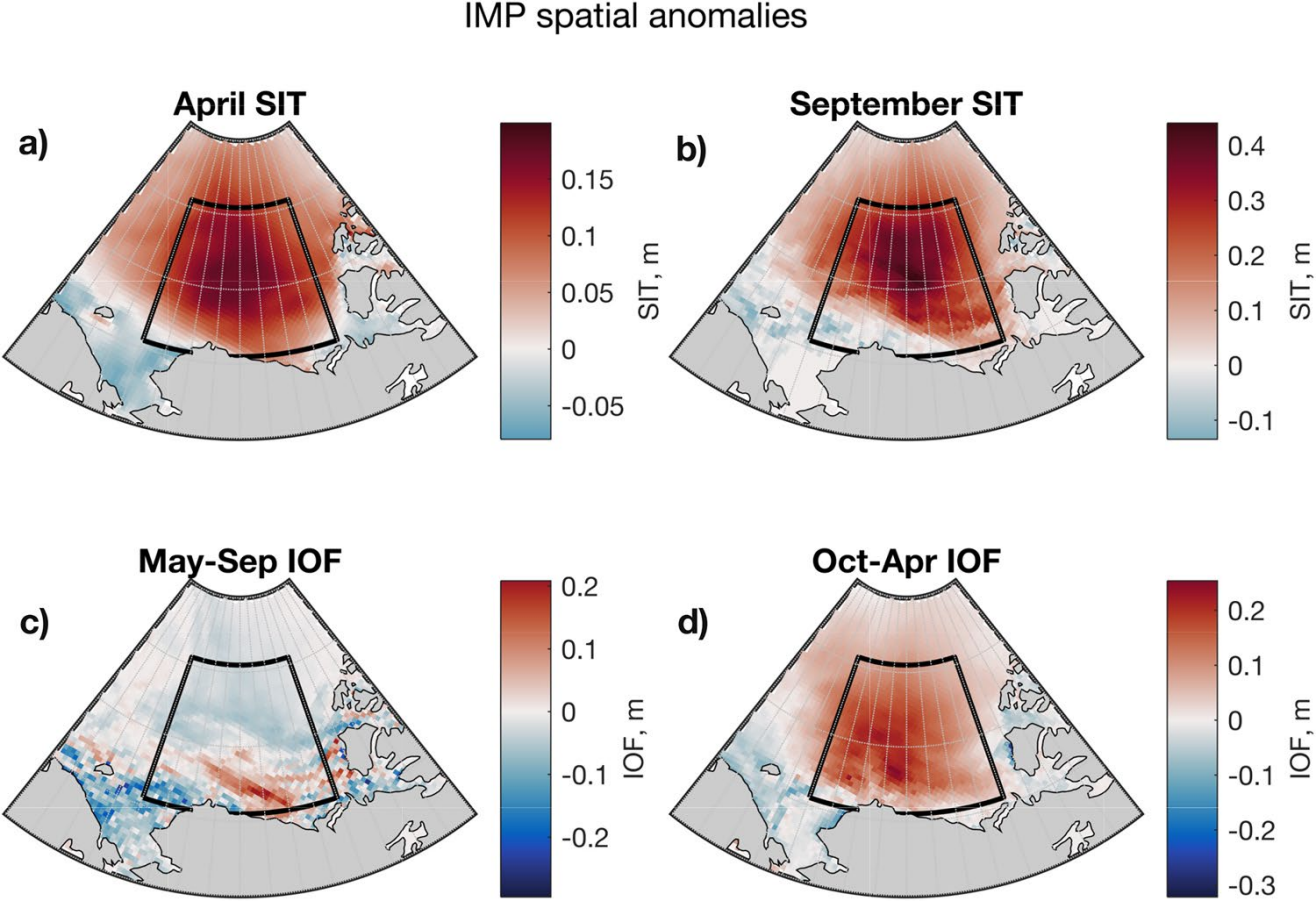
Seasonal evolution of anomalies in ice thickness and ice thermodynamics in IMP (defined in Table 2). Red colours in **a**, **b** denote a thicker sea ice cover in April and September, respectively. Blue colours in **c** indicate decreased summertime melting, while red colours in **d** show decreased wintertime freezing

In contrast, the difference between the BGM10 anomalies and BGM anomalies yields an ice import scenario, IMP (Fig. 8). The anticlockwise rotation of the cyclonic wind anomaly causes ice convergence and yields a dynamically thickened sea ice anomaly in the gyre region in IMP. In the summertime, this thick sea ice anomaly preconditions enhanced melting in the southern part of the gyre region (Fig. 8a,c), where ice generally melts by the end of summer (c.f. Fig. 4c); increased ice thickness provides increased freshwater availability. Towards the basin interior, the thick anomaly leads to lower summer ice melt (Fig. 8c).

During winter, the thick anomaly suppresses sea ice growth (Fig. 8b, d). The spatial correlation between the mean September sea ice thickness anomaly and the mean winter ice-ocean freshwater flux is $R=0.76$. The strong winter signal in the ice-ocean freshwater flux dominates over the spatially heterogeneous summer pattern, such that the net annual effect of relative sea ice import is one of increased freshwater flux from the ice to the ocean.

## Concluding discussion

In this paper, we have investigated how the interplay between mechanically driven sea ice transport and thermodynamic sea ice growth rate affects the mean state and variability of Beaufort Gyre liquid freshwater content. Using forced experiments with the MITgcm, we have shown that BG FWC is sensitive to the degree of ice import/export into the region. Specifically, increased sea ice export yields reduced FWC in the gyre, while increased sea ice import yields increased FWC in the gyre. The same mechanism appears to be at play in three other ice-ocean models, and—because these models exhibit different responses of sea ice transport to the same imposed wind forcing patterns—is a source of variability that may help to explain the spread in BG FWC between the CRFs presented here (Fig. 3), and other model simulations more generally.

Using the MITgcm, we have isolated the effect of anomalies in ice transport and examined spatial and seasonal responses to these anomalies. Sea ice export leads to thinning of the mean sea ice cover in the region (Fig. 7a, b), promoting sea ice growth during winter (Fig. 7d), because the thermodynamic growth rate of ice is inversely related to its thickness (e.g. Thorndike et al. 1975; Bitz and Roe 2004). During summer, dynamic thinning yields a spatially varying response. There is increased melting in the interior of the Canada Basin, most likely due to earlier and increased penetration of solar radiation into the mixed layer, warming the ocean (Perovich et al. 2008, 2011; Steele and Dickinson 2016). Meanwhile, there is decreased melting along the southern margin of the region (Fig. 7c) due to reduced ice availability in this region where ice rarely survives the summer in the model (Fig. 4) and in observations (Stroeve et al. 2011). The net effect of these seasonally distinct responses is a reduction of BG FWC under ice export. In the other case, with anomalous sea ice import, the pack ice thickens (Fig. 8a, b), winter freezing reduces in intensity (Fig. 8d), and summer melting reduces across the interior and increases on the southern margin (Fig. 8c). The winter signal dominates, leading to increased FWC in the BG. This explanation is consistent with the export case—the patterns are opposite, but the underpinning physical relationships are the same. Our results also highlight the importance of the cross-isobaric angle (ageostrophic component) of the surface wind for sea ice dynamics in the BG, and—via the mechanism outlined above—FWC.

Previous work has explored the interplay between a source of freshwater from Ekman convergence in the upper ocean (modulated by the ice-ocean governor effect), and a sink due to eddy fluxes (e.g. Davis et al. 2014; Manucharyan and Spall 2016; Doddridge et al. 2019). Here, we identify a further pathway for freshwater due to ice transport and compensating thermodynamic responses. The impact of this term on the FWC reservoir is buffered by the adjustment of FWC-dependent sinks (Manucharyan et al. 2016), explaining why anomalies in the cumulative flux of freshwater between ice and ocean are larger in magnitude than resulting anomalies in FWC (Figs. 5, 6). Our results also show that BG flushing or accumulation events may be enhanced in magnitude due to the identified mechanism (depending on the cross-isobaric angle of winds and ice flow in the gyre region). To the extent that the mechanism modifies freshwater fluxes into the subpolar North Atlantic, it may be consequential for the strength of the AMOC (Jahn and Holland 2013; Yang et al. 2016; Wang et al. 2018a; Holliday et al. 2020).

Our findings have both strengths and caveats. In coordinated experiments with four ice-ocean models, we found evidence that a compensation existed between anomalies in sea ice export/import, and thermodynamic growth rate: sea ice volume anomalies were limited in all cases. In the BGP experiments, differences in sea ice transport likely explain some of the inter-model spread in FWC accumulation (Fig. 3). However, without further experiments, it was not possible to isolate the effect on FWC due to sea ice transport. Using the MITgcm, we isolated the impact of changing sea ice transport by performing targeted experiments with modified wind fields. We were further able to assess the seasonal and spatial characteristics of the processes at play. We used a CRF approach, applying a stationary wind perturbation for 34 years. However, satellite observations show that sea ice transport anomalies into the BG also exhibit substantial variability on interannual timescales (e.g. Mallett et al. 2021; Stroeve et al. 2011). Further experiments could probe the impact of short-lived and high magnitude sea ice transport anomalies on the FWC of the BG. Additionally, further experiments could investigate how dependent the details of the outlined mechanism are on the choice of model, or indeed on climate conditions—would the same relationships hold in a warmer climate?

One caveat to extrapolating our model-based results to nature comes from our use of ice-ocean models. These models enable us to apply specific atmospheric forcings, but naturally do not represent atmospheric feedbacks. A potentially important feedback results from changing ice growth rate and corresponding vertical heat fluxes across sea ice, which can change the surface air temperature (e.g. Maykut 1978). However, surface air temperatures are positively correlated with ice growth in coupled climate models in the contemporary and historical Arctic (Petty et al. 2018), suggesting that this atmospheric feedback has not been an important limitation on ice growth. Examining the mechanism outlined here in coupled climate models would be a natural next step to test the importance of such feedbacks.

Our targeted experiments with the MITgcm highlight the importance of angular relationships in the air-sea ice boundary layer in affecting Beaufort Gyre FWC. Under a stationary SLP pattern, we have seen that (a) changing the cross-isobaric angle of surface winds by 10$^\circ$ can affect FWC under a naturally varying climatology (Fig. 5); (b) depending on the cross-isobaric angle of surface winds, responses of FWC to wind forcing are different (Fig. 6). Observations show a wide range and strong seasonal cycle in the cross-isobaric angle of surface wind (e.g. Maeda et al. 2020; Kimura and Wakatsuchi 2000). We suggest that uncertainties in this angle, and in the flow directions of sea ice in response to surface winds, should be considered important for large-scale hydrographic properties and ocean dynamics. Significant natural variability exists in the angular relationships across the polar marine and atmospheric boundary layers (Cole et al. 2017; Maeda et al. 2020; Heorton et al. 2019); accurately representing these boundary layer processes in climate models is a significant challenge (e.g. Hunke et al. 2011). Our results provide further large-scale motivation for improving the representation of these relatively small-scale processes.

## Data Availability

The datasets analysed in this paper are available in the Arctic Data Center repository, Beaufort Gyre Climate Response Function experiments: sea ice transport and freshwater content, 10.18739/A20K26C63.
